# Mobile Tigecycline Resistance: An Emerging Health Catastrophe Requiring Urgent One Health Global Intervention

**DOI:** 10.3389/fmicb.2022.808744

**Published:** 2022-08-01

**Authors:** Madubuike Umunna Anyanwu, Obichukwu Chisom Nwobi, Charles Odilichukwu R. Okpala, Ifeoma M. Ezeonu

**Affiliations:** ^1^Microbiology Unit, Department of Veterinary Pathology and Microbiology, Faculty of Veterinary Medicine, University of Nigeria, Nsukka, Nigeria; ^2^Department of Veterinary Public Health and Preventive Medicine, Faculty of Veterinary Medicine, University of Nigeria, Nsukka, Nigeria; ^3^Department of Functional Food Products Development, Faculty of Biotechnology and Food Science, Wrocław University of Environmental and Life Sciences, Wrocław, Poland; ^4^Department of Microbiology, Faculty of Biological Sciences, University of Nigeria, Nsukka, Nigeria

**Keywords:** health threat, mobile tigecycline resistance, One Health, tetracycline overuse, antimicrobial resistance

## Abstract

Mobile tigecycline resistance (MTR) threatens the clinical efficacy of the salvage antibiotic, tigecycline (TIG) used in treating deadly infections in humans caused by superbugs (multidrug-, extensively drug-, and pandrug-resistant bacteria), including carbapenem- and colistin-resistant bacteria. Currently, non-mobile *tet*(X) and mobile plasmid-mediated transmissible *tet*(X) and resistance-nodulation-division (RND) efflux pump *tmexCD-toprJ* genes, conferring high-level TIG (HLT) resistance have been detected in humans, animals, and environmental ecosystems. Given the increasing rate of development and spread of plasmid-mediated resistance against the two last-resort antibiotics, colistin (COL) and TIG, there is a need to alert the global community on the emergence and spread of plasmid-mediated HLT resistance and the need for nations, especially developing countries, to increase their antimicrobial stewardship. Justifiably, MTR spread projects One Health ramifications and portends a monumental threat to global public and animal health, which could lead to outrageous health and economic impact due to limited options for therapy. To delve more into this very important subject matter, this current work will discuss why MTR is an emerging health catastrophe requiring urgent One Health global intervention, which has been constructed as follows: (a) antimicrobial activity of TIG; (b) mechanism of TIG resistance; (c) distribution, reservoirs, and traits of MTR gene-harboring isolates; (d) causes of MTR development; (e) possible MTR gene transfer mode and One Health implication; and (f) MTR spread and mitigating strategies.

## Introduction

Antimicrobial resistance (AMR) continues to be a growing threat to the global public health (Silvester et al., 2021). Due to the promiscuous nature of plasmids, the resistance mechanism (mediated by a plasmid) are understood to spread horizontally/laterally among bacteria with lightning speed. It is no longer news in medicine that globally, estimated human deaths due to infections by superbugs (multi-, extensively-, and pandrug-resistant bacteria), including carbapenem- and colistin-resistant bacteria, stand at about 700,000, eventually projected to reach 10 million by 2050 (O’Neill, 2014). Each year across Europe and the US alone, superbugs are responsible for the loss of more than 50,000 lives (O’Neill, 2014). In Africa, AMR-related annual deaths by 2050 are poised to reach about 4,000,000 (O’Neill, 2014). AMR is a One Health global problem that imposes enormous financial burden on countries irrespective of income level (Collignon et al., 2018).

By 2030, AMR could force up to an estimate of 24 million people into extreme poverty (World Health Organization [WHO], 2019) and by 2050, it could cause a global loss of US$100 trillion, which would decrease the (global) domestic product by 3.5% (O’Neill, 2014). In US alone, the challenge of AMR causes direct healthcare costs of about US$20 billion, excluding annual productivity loss of about US$35 billion (Dagostar, 2019). Developing countries [that is low-and middle-income countries (LMIC) in Asia, Africa and South America] remain the development/emergence hotspots of AMR—characterized by poor health infrastructure, poor hospital seeking habit, inadequate antimicrobial stewardship programs, unrestricted access to antibiotics, poor record-keeping, and weak or poor governance and regulatory framework (Ayukekbong et al., 2017; Anyanwu et al., 2021a). Accordingly, the AMR impacts greatly on poor nations that lack disease prevention and affordable treatments facilities (Silvester et al., 2021). For example, Thailand has estimated that superbugs are associated with respective annual death and economic loss of over 38,000 people and US $1.3 billion (Thamlikitkul et al., 2015). Traveling and trade facilitates the dissemination of AMR; thus the increasing population of most LMICs coupled with war and unstable governments, cause huge migration of the people thereby making it easy for resistant organisms to spread from these regions to other parts of the world (Yam et al., 2019; Bunduki et al., 2020).

Despite the increasing rate of development and spread of plasmid-mediated resistance against the two last-resort antibiotics, colistin (COL) and tigecycline (TIG), it is terrifying that there is no new safe and effective antimicrobial class coming to the market at least in the foreseeable future (Theuretzbacher et al., 2020). Essentially, this situation of emergence and spread of plasmid-mediated high-level TIG (HLT) resistance is very crucial and must not be taken for granted. Indeed, there is need to alert the global community to increase their antimicrobial stewardship, particularly the developing nations. Justifiably, MTR spread projects One Health ramifications and portends a monumental threat to global public and animal health, which could lead to outrageous health and economic impact due to limited options for therapy. To delve more into this very important subject matter, this current work will discuss why MTR is an emerging health catastrophe requiring urgent One Health global intervention, which has been constructed as follows: (a) antimicrobial activity of TIG; (b) mechanism of TIG resistance; (c) distribution, reservoirs, and traits of MTR gene-harboring isolates; (d) causes of MTR development; (e) possible MTR gene transfer mode and One Health implication; and (f) MTR spread and mitigating strategies.

## Antimicrobial Activity of Tigecycline

Tigecycline is a third-generation tetracycline (belonging to glycycline class/category) antibiotic considered a salvage drug for treating deadly infections caused by superbugs, including carbapenem- and COL-resistant bacteria in humans. It was the first glycycline derived in the 1990s from the semisynthetic minocycline in an effort to overcome TET resistance (Zhanel et al., 2004). TIG has strong antibacterial activity against most Gram-positive and Gram-negative bacteria except *Proteus* and *Pseudomonas* (Yu R. et al., 2021), and it is approved only for treating complicated skin and skin structure infections (cSSTI) with the exclusion of diabetes foot infection, complicated intra-abdominal infections (cIAI) and community-acquired pneumonia (CAP) (Chen et al., 2020; Cui et al., 2020; Yaghoubi et al., 2021). TIG inhibits bacterial protein synthesis by reversibly binding to the 16S rRNA, hindering amino-acyl tRNA molecules from entering the A site of the ribosome and inhibiting the elongation of peptide chains (Zhang et al., 2020a; Yaghoubi et al., 2021). Chemical modification of TIG at the C-9 position of ring D led to enhanced binding to the target and more effective evasion of common TET resistance mechanisms, when compared to older TETs (tetracycline, doxycycline, and minocycline) (Zhang et al., 2020a).

First-generation TETs consisting of tetracycline, chlortetracycline and oxytetracycline were put into clinical practice in 1952 (Nelson and Levy, 2011) while the second-generation TETs made up of doxycycline and minocycline were put into use in 1976 (Fang et al., 2020). These antibiotics have been incorporated into animal feed to improve growth and feed efficiency (Zhang et al., 2021). They exert a bacteriostatic effect (inhibiting bacterial protein synthesis) against a wide variety of Gram-positive and Gram-negative bacteria as well as atypical/intracellular organisms such as chlamydiae, mycoplasmas, and rickettsiae, and protozoan parasites by binding reversibly to the bacterial 30S and to some extent 50S ribosomal subunit and blocking incoming aminoacyl tRNA from binding to the ribosome acceptor site (Chopra and Roberts, 2001; Roberts, 2003). In addition, they could alter the cytoplasmic membrane causing intracellular components to leak from bacterial cells (Roberts, 2003). Although approved for human medicine by the United States FDA in 2005 and then in Europe in 2006 (Aminov, 2013) and China in 2010 (Zhang et al., 2020a), TIG has never been approved/used in livestock husbandry anywhere around the globe (He et al., 2019; Cui et al., 2020).

## Mechanism of Tigecycline Resistance

TIG resistance similar to older tetracyclines, particularly before 2013, was understood as mutationally-acquired and chromosomally-encoded, as mediated by overexpression of chromosomal efflux pump regulator genes such as *ramAR*, *acrAB*, *OqxR*, and others (Sun S. et al., 2020; Wang et al., 2021b; Yaghoubi et al., 2021). The *tet*(X)—a gene encoding tetracycline destructases considered flavin-dependent monooxygenases [Class A flavin monooxygenases (FMOs) requiring oxygen to function/confer resistance], capable of degrading all TETs, including TIG, as well as the recently FDA-approved fourth-generation TETs eravacycline and omadacycline, was deemed chromosomally-mediated and vertically transmitted only among the environmental and commensal microbiota (Sun J. et al., 2019; Fang et al., 2020; Aminov, 2021; Zhang et al., 2021). Therefore, TIG resistance might pose no serious risk because pathogenic organisms have not evolved *tet*(X) and the chromosome-mediated mechanisms, including chromosome-borne *tet*(X) orthologs (genes)—*tet*(X), *tet*(X1), and *tet*(X2), are only vertically transmitted, conferring resistance to low-level TIG and thus self-limiting by their very nature. Structurally, Tet(X) comprises a substrate-binding domain, coenzyme flavin adenine dinucleotide (FAD)-binding domain and C-terminal α-helix. Site-directed mutagenesis confirmed that the resistance phenotype/activity (capacity to mediate low-, moderate-, or high-level TIG resistance) of *tet*(X) variants is dependent on the residues in the TETs binding region and sites in the FAD binding region (Cui et al., 2021a,b,c). Thus, the accumulation of beneficial mutations results in increased activity (ability to exert moderate or HLT resistance) of *tet*(X) variants (Cui et al., 2021a). The *tet*(X1) is inert and inactive, but non-self-transmissible chromosome-borne *tet*(X2) possibly with accumulated beneficial mutations was recently shown to confer a rare HLT resistance (MIC ≥ 16 mgL^–1^) only in *Flavobacteriaceae*, believed to be the ancestral source of *tet*(X) gene (Zhang et al., 2020a; Cui et al., 2021a,c). The knowledge about the active sites that lead to high-level resistance and about the evolutionary path of Tet(X) remains limited (Cui et al., 2021c). Nevertheless, a Sierra Leone study conducted in 2013 detected the *tet*(X) in a high proportion (21% of 52) of human clinical isolates belonging to diverse families (Leski et al., 2013). This finding signaled that *tet*(X) has been acquired by pathogenic bacteria probably from commensal/environmental microbiota through plasmid-mediated horizontal gene transfer (HGT) (Aminov, 2021). Hence, the increasing research interest in TIG resistance conferred by the *tet*(X) gene.

Unfortunately, as the globe grappled with the plasmid-mediated mobile COL resistance (*mcr*) gene [which had been discovered in the late 2015 and able to confer resistance to colistin (COL), a last resort antibiotic], in May 2019, the plasmid-encoded transmissible *tet*(X), *tet*(X3), and *tet*(X4) genes conferring HLT resistance were discovered in bacterial isolates from food animals, meat and environment in China (He et al., 2019). This discovery suggested the clinical efficacy of TIG is being threatened by the mobile TIG resistance (MTR). Retrospective analysis showed that MTR possibly emerged in 2016, thus there is speculation it is a recent event unlike *mcr* that evolved in the 1980s but detected approximately 40 years later (Sun C. et al., 2019). Nevertheless, the paradigm version of *tet*(X), *tet*(X0) gene was discovered on an R plasmid from a *Bacteroides fragilis* isolate (an anaerobe hence no effect on it) as early as the 1980s suggesting that plasmid-encoded *tet*(X) is not a recent event (Cui et al., 2021a). Since the discovery of mobile *tet*(X) genes in China, other reports are rapidly emerging regards the diverse ecological niches worldwide (Ding et al., 2020; Fang et al., 2020; Pan et al., 2020; Aminov, 2021; Marathe et al., 2021; Mohsin et al., 2021). Currently, *tet*(X) genes (both mobile and non-mobile) have been detected in at least 30 countries in 6 continents except Antarctica (Ding et al., 2020; Pan et al., 2020; Mohsin et al., 2021; Soliman et al., 2021; Usui et al., 2021; Wang et al., 2021f). To worsen the situation, barely few months (in April 2020) after the discovering of mobile *tet*(X) genes, a plasmid-encoded transmissible resistance-nodulation-division (RND) efflux pump gene, *tmexCD1-toprJ1* also conferring HLT resistance, was discovered in *Klebsiella pneumoniae* isolates from chickens in China (Lv et al., 2020). However, the mechanistic evolution of MTR is yet to be fully understood (Yongchang et al., 2019). Notably, very recently, plasmid-mediated TIG resistance-related gene, *tet*(Y) causing a decreased TIG susceptibility and transmitted across isolates by transposable element Tn*5393*, was detected in a clinical *Acinetobacter baumannii* isolate from China (Wang et al., 2021i). Additionally, mutations in plasmid-encoded *tet*(A) and *tet*(L) caused increased TIG resistance in virulent *K*. *pneumoniae* under selective pressure (Zhang et al., 2020a; Xu et al., 2021; Sun et al., 2022). The rapid transfer and acquisition of mobile *tet*(X) and *tmexCD1-toprJ1* genes is because they are carried by highly promiscuous mobile genetic elements like plasmids containing transposons, insertion sequences, and integrons (Aminov, 2021; Cui et al., 2021a,b). Thus, with MTR fast-spreading globally, it poses a monumental threat to antimicrobial therapy since TIG is a last-ditch drug for treating deadly infections in humans. Sadly, there remains this dilemma that not one specific antimicrobial can be used to treat the deadly infections especially those associated with TIG-resistant (TIG-r) bacteria. These organisms are potentially pandrug-resistant, so infections caused by them often result in fatality. Hence, we put to light the danger posed by the rapidly emerging and spreading plasmid-mediated HLT resistance and the need for the global community to halt injudicious use of TETs/TIG to protect global public health.

## Distribution, Reservoirs and Traits of Mobile Tigecycline Resistance Gene-Harboring Isolates

Ecological niches in which plasmid-encoded TIG resistance *tet*(X) genes have been detected are shown in Table 1. Clearly, there is increasing evidence of plasmid-encoded TIG resistance *tet*(X) genes reported in countries across Europe, Asia, United States, South America, and Africa involving humans, food animals, including chickens, pigs, ducks, pigeons, geese, and cattle, as well as companion animals, food animal products (meats), aquaculture and environment such as soil, livestock-farm water, wastewaters, sewages, and wildlife as reservoirs of MTR (Bai et al., 2019; Chen et al., 2019a,b, 2020, 2021; He et al., 2019; Sun C. et al., 2019, 2020; Sun J. et al., 2019; Cao et al., 2020; Cui et al., 2020, 2021b; Ding et al., 2020; Du et al., 2020; He T. et al., 2020; Li et al., 2020, 2021f; Ma et al., 2020; Pan et al., 2020; Ruan et al., 2020; Song et al., 2020; Wang et al., 2020a,b; Zhang et al., 2020b, 2021; Zheng et al., 2020; Cheng et al., 2021a,b; Feng et al., 2021; Hirabayashi et al., 2021a,b; Hsieh et al., 2021; Li et al., 2021e,f; Lu et al., 2021; Marathe et al., 2021; Martelli et al., 2021; Mohsin et al., 2021; Soliman et al., 2021; Sun et al., 2021a,b; Tang et al., 2021; Wang et al., 2021f,g; Xu et al., 2021; Yu Y. et al., 2021; Wu et al., 2022; Zhai et al., 2022). We further demonstrate this, through the specific reservoirs in which plasmid-borne transmissible *tet*(X) and *tmexCD-toprJ* genes have been detected as shown in Table 2. Publications involving *tet*(X) genes appear to be more in humans, chickens and pigs, whereas those for *tmexCD-toprJ* genes appear to be more in humans and chickens. It is important to reiterate that from these ecological niches, the various MTR genes, such as *tet*(X3/3.2), *tet*(X4), *tet*(X5/5.2/5.3), *tet*(X6), *tet*(X7), and *tet*(X18), have been detected in a diversity of bacteria, which include *Escherichia*, *Rauoltella*, *Myroides*, *Enterobacter*, *Citrobacter*, *Klelbsiella*, *Shingobacterium multivorum*, *Proteus*, *Acinetobacter*, *Salmonella*, *Riemerella anatipestifer*, and *Empedobacter brevis* (Bai et al., 2019; He et al., 2019; Sun C. et al., 2019; Yongchang et al., 2019; Li et al., 2020, 2021a,c,d,^2022^; Liu et al., 2020; Wang et al., 2020a, 2021f; Zhang et al., 2020a,b; Chen et al., 2021; Cheng et al., 2021a,b; Soliman et al., 2021; Xu et al., 2021). Notably, sequence alignment results revealed that *tet*(X5.2/5.3) genes shared 96.3–100% nucleotide sequence identity with *tet*(X6) and its variant (Chen et al., 2020; He D. et al., 2020; Zheng et al., 2020).

**TABLE 1:** Ecological niches in which plasmid-encoded tigecycline resistance *tet*(X) genes have been detected.

Region	Country	Source of mobile *tet*(X) gene-harboring organism	Mobile *tet*(X) gene detected	*tet(X)*-carrying organism	*tet(X)*-associated plasmid	Additional resistance factors	Sequence types (Virulence genes/phylogrooup)	Contain genes encoding resistance against other last-resort antimicrobials	References
Asia	Japan	Pig	*tet*(X6)	*E*. *coli*	IncW	*aadA1*, *aadA2*, *cmlA1*, *dfrA1*, *sul1*, and *sul3*, Tn*As3*, and IS*Vsa3*	ST9222 (*capU*, *fyuA*, *gad*, *iha*, *sitA*, *terC*, and *traT*)	No	Usui et al., 2021
	Vietnam	Urban drainage	*tet*(X)	*Klebsiella aerogenes*	IncC-IncX	*bla*_NDM–4_, *bla*_CTX–M–14_, *qnrS1*, *aac(6′)-lb-cr*, *cfr*, IS*26*, and IS*Vsa3*	−	Yes	Hirabayashi et al., 2021a
	Taiwan, China	Humans	*tet*(X6)	*Acinetobacter baumannii*	112 kb CMCVTAb1-Ab59 and 9 kb pAB-NCGM253	*bla*_OXA–72_, *bla*_OXA–66_, *ant(30)-IIa*, *aph(30)-Ib*, *aph(6)-Id*, *aph(30)-Ia*, *tet(Y)*, *abaQ*, ΔIS*CR2*, IS*CR2*, IS*26*, and Tn3	ST793 and ST723	Yes	Hsieh et al., 2021
	Singapore	Humans	*tet*(X4)	*E*. *coli*, *K*. *pneumoniae* and *Enterobacter clocae*	IncI1	***mcr*-1**, *bla*_TEM–1_, *bla*_CTX–M65_, *aac(3)-IV*, *aph-(4)-Ia*, *floR*, *fosA*, IS*CR2*, and ΔIS*CR2*	ST515 and others	Yes	Ding et al., 2020
	Pakistan	Human, chickens, chicken meat, and environment (wild bird and slaughterhouse wastewater)	*tet(X4)*	*E*. *coli*	IncFII, IncHI2 and IncQ	***mcr*-1**, *aadA1*, *aadA2*, *aadA8*, *aph(3’)-lb*, *aph(6)-ld*, *aph(3′)-Ia*, *aph(3″)-Ib*, *aadA22*, *aadA5*, *aac(3)-lle*, *aac(6′)-Ib-cr*, *aadA5*, *aac(3)-IId*, *arr-2*, *bla*_EC–18_, *bla*_EC–15_, *bla*_TEM–215_, *bla*_TEM–1B_, *bla*_TEM–215_, *bla*_CMY–2_, *bla*_CTX–M–15_, *bla*_TEM–176_, *bla*_TEM–1_, *bla*_OXA–1_, *cmlA1*, *dfrA1*, *dfrA12*, *arr-2*, *dfrA14*, *dfrA15*, *dfrA17*, *floR*, *osA3*, *fosA4*, *mph(A)*, *qnrS1*, *sul1*, *sul2*, *sul3*, *tet(A)*, *tet(B)*, *qnrS1*, *qnrS2*, *lnu(F)*, *mdf(A)*, *sul1*, IS*CR2*, and ΔIS*CR2*	ST6726 and ST224, ST6751, ST351, ST694, ST69, ST155, ST2253, ST2099, ST189, ST6706, ST1638, ST2847, ST2207, ST2234, ST48, and ST410 (*astA*, *cea*, *cma*, *capU*, *gad*, *hra*, *iss*, *iucC*, *iutA*, *lpfA*, *neuC*, *ompT*, *papA_F19*, *sitA*, *gad*, *terC*, and *traT*)	Yes	Li et al., 2021a,b,c,d; Mohsin et al., 2021
	China	Humans, food animals (cattle, chickens, pigs, geese, pigeons, and ducks), food-animal products (pork, beef, and chicken meat), environment (farm water, soil, sewage, slaughterhouse environment, animal farm environment, and migratory wild birds), aquatic/aquaculture system (shrimps)	*tet*(X3), *tet*(X4), *tet*(X5), *tet*(X5.2, *tet*(X5.3), *tet*(X6), *tet*(X7), and *tet*(X18)	*Escherichia, Klebsiella pneumoniae*, *Acinetobacter*, *Proteus*, *Salmonella*, *Citrobacter*, *Enterobacter cloacae*, *Rauoultella ornithinolytica, Riemeriella*, *Empedobacter*, and *S. multivorum*	IncX, IncX1, IncX4, IncI2, IncQ1, IncFIB, IncFII, IncFI, IncFIA, IncHIA ColE. ColRNA1, and IncHIB IncHI1A, IncHI1B, IncA/C2, IncX1-IncN, IncX1-IncR, IncX1-IncFIA/B-IncY, IncX1-IncFIA/B-IncHI1A/B IncFIA/B-IncHI1A/B, IncFIA18- IncFIB-IncX	***mcr*-1**, *bla*_NDM–58_, *bla*_NDM–5_, *bla*_NDM–1_, *bla*_TEM–1C_, *bla*_TEM–1A_, *bla*_TEM–290_, *bla*_SHV–12_, *bla*_OXA–282_, *bla*_CTX–M–14_, *bla*_TEM–30_, *bla*_CTX–M–65_, *bla*_CTX–M–27_, *bla*_CTX–M–122_, *bla*_TEM–150_, *bla*_OXA–10_, *bla*_OXA–83_, *bla*_LAP–2_, *bla*_CMH–3_, *bla*_EBR–1–like_, *bla*_CTX–M–55_, *bla*_TEM–1B_, *bla*_OXA–58_, *bla*_CMY2_, *bla*_DHA–1_, *ble*, *bla*_CARB_, *bla*_CARB–2_, *qacE1*, *qacL*, *erm*(42), *erm*(B), *lnu*(G), *lnu*(F), *qnrS2, qnrS1, qnrVC6*, *qnrD*, *oqxA*, *oqxB*, *strA*, *strB*, *aac(6′)-Ib*, *aac(6′)-Ib-cr*, *ant(3”)-Ia*, *aac(3)*, *aph*, *aadA2, aadA22, aadA1, aac(3)-IId, aph(3′)-Ia, acc(3)-Iva*, *aph(4)-Ia*, *aac(3)-IIa*, *aph(3′)-Ib*, *ant(3′)-Ih*, *aph(6”)-Id*, *aph(3′)-Via*, *aac(3)-Via*, *qacE1*, *aadS, arr-3, dfrA14, dfrA1*, *dfrA12*, *dfrA3b*, *dfrA20*, *dfrA5*, *dfrA16*, *cmlA1*, *cmlA6*, *cmlB1*, *cat*, *catB8*, *catB3*, *cfr*, *floR, fosA, mph*(A), *mph*(E), *msr*(E), *mef*(B), *mdf*(A), *sul1*, *sul2*, *sul3*, *tet*(A), *tet*(B), *tet*(M), *tet*(H), *tet*(Y), *tet*(J), *copA-D*, *cusR/S*, *czcA-D*, class 1 integrase *IntI*1, Tn*6451*, TnpA; IS*Vsa3*, IS*CR2*, IS*26*, IS*Aba1*, IS*4351*, IS*1R* and IS*1380*	ST3997, ST2325, ST3944, ST1421, ST515, ST10120, ST761, ST206, ST847, ST877, ST773, ST8302, ST871, ST10170, ST4656, ST2035, ST10392, ST4429, ST1602, ST10, ST744, ST761, ST746, ST2345, ST8504, ST189, ST1437, ST112, ST515, ST10115, ST641, ST1196, ST1638, ST10671, ST195, ST101, ST4704, ST6775, ST218, ST6833, ST2144, ST795, ST58, ST284, ST215, ST165, ST2065, ST789, ST540, ST1196, ST2064, ST642, ST1308, ST4156, ST295, ST761, ST34, ST278, ST453, ST6704, ST4541, ST972, ST7450, ST410, ST767, ST29 and ST48 (*irp2*, *fyuA*, *ybtA*, *astA*, *espX4*, *espX5*, *espY1*, *fik iuc*, *ybT*, and *ibeB/*A, B1and E) *K*. *pneumoniae*: ST37, ST534 and ST1418 *Salmonella*: ST8300 *Acinetobacter baumanii*: ST355 (*silP*)	Yes	Bai et al., 2019; Chen et al., 2019a,b, 2021; He et al., 2019; Sun J. et al., 2019; Sun C. et al., 2019; Cao et al., 2020; Chen et al., 2020; Cui et al., 2020; Du et al., 2020; He T. et al., 2020; Li et al., 2020; Ma et al., 2020; Pan et al., 2020; Ruan et al., 2020; Song et al., 2020; Sun S. et al., 2020; Wang et al., 2020a,b; Zhang et al., 2020a,b; Zheng et al., 2020; Cheng et al., 2021a,b; Cui et al., 2021b; Feng et al., 2021; Li et al., 2021a,c,d,e,f; Lu et al., 2021; Sun et al., 2021a,b; Tang et al., 2021; Wang et al., 2021c,f,g; Xu et al., 2021, Yu Y. et al., 2021; Zhang et al., 2021; Wu et al., 2022; Zhai et al., 2022
Africa	Egypt	Chickens	*tet*(X7)	*E*. *coli*	IncHI2, IncHI2A, IncFIA, IncFIB, and IncFIC (FII)	***mcr*-1.1**, *erm(B)*, *aph(3″)-Ib*, *aph(6)-Id*, *aac(3)-IIa*, *aph(3′)-Ia*, *aadA1*, *sul1*, *fosA*, *sul3*, *dfrA7*, *dfrA14*, *dfrA1*, *arr-2*, *bla*_CTX–M–15_, *bla*_TEM–1B_, *mph(A)*, *ere(A)*, *erm(B)*, *tet(A)*, *cmlA1*, and *floR*	ST155 and ST10 (*sitABCD*, *iucABCD*, and *iutA*)	Yes	Soliman et al., 2021
Europe	Norway	Wastewater	*tet*(X4)	*E*. *coli*	IncFII and IncI1-I and IncX4	*bla*_CTX–M–14_, *erm(B)*, *erm(42)*, *qnrS1*, *dfrA12*, *sul2*, *tet(M)*, *aph(6)-Id*, *aadA1*, *aadA2*, *ant(2″)-Ia*, *aph(3″)-Ib*, and *cml*	ST167	Yes	Marathe et al., 2021
	United Kingdom	Pig	*tet*(X4)	*E*. *coli*	IncX1-IncY	*bla*_TEM–1b_, *floR*, *aadA2*, and *linG*	ST1140	No	Martelli et al., 2021

*Sequence type (ST): Warwick multilocus sequence type of tet(X)-positive E. coli isolates except otherwise stated; ST for Acinetobacter baumannii is based on Pasteur and Oxford schemes.*

**TABLE 2:** Specific reservoirs in which plasmid-borne transmissible *tet*(X) and *tmexCD-toprJ* genes have been detected.

Reservoir	References	
	*tet*(X)	*tmexCD-toprJ*
Humans	He et al., 2019; Wang et al., 2019, 2020a,b,2021f,2021g; Chen et al., 2020; Ding et al., 2020; Pan et al., 2020; Zhang et al., 2020a; Cui et al., 2021b; Hsieh et al., 2021; Li et al., 2021a,b; Zeng et al., 2021	Lv et al., 2020; He et al., 2021; Hirabayashi et al., 2021a; Qin et al., 2021; Wang et al., 2021b,h; Yang et al., 2021; Liu et al., 2022; Xiao et al., 2022; Zhai et al., 2022
Chickens	He et al., 2019; Li et al., 2019; Sun J. et al., 2019; Sun C. et al., 2020; Zheng et al., 2020; Chen et al., 2021; Fu et al., 2021; Li et al., 2021c,d,f; Mohsin et al., 2021; Sun et al., 2021a; Wang et al., 2021a; Xu et al., 2022	Lv et al., 2020; Sun S. et al., 2020; Li et al., 2021e; Wang et al., 2022; Xu et al., 2022
Pigeons	Cui et al., 2020; Wang et al., 2020b; Lu et al., 2022	−
Ducks	Cui et al., 2020; Ji et al., 2020; Pan et al., 2020; Wang et al., 2020b; Sun et al., 2021a; Yu Y. et al., 2021	−
Geese	Cui et al., 2020; Ji et al., 2020; Wang et al., 2021g	
Pigs	Bai et al., 2019; He et al., 2019; Sun C. et al., 2019; Sun J. et al., 2019; Chen et al., 2020; He T. et al., 2020; Li et al., 2020; Ma et al., 2020; Sun S. et al., 2020; Song et al., 2020; Cheng et al., 2021a,b; Feng et al., 2021; Fu et al., 2021; Martelli et al., 2021; Peng et al., 2021; Tang et al., 2021; Usui et al., 2021; Wang et al., 2021c; Xu et al., 2021; Li et al., 2022; Wu et al., 2022	Peng et al., 2021
Cattle	He et al., 2019; Zhang et al., 2020b; Fu et al., 2021	−
Companion animals	Sun et al., 2021b	−
Meat	Bai et al., 2019; Du et al., 2020; Li et al., 2021c,e; Sun et al., 2021a	Peng et al., 2021
Aquatic food	Li et al., 2019	−
Soil	Cao et al., 2020; Ji et al., 2020; Li et al., 2020, 2021f; Yu Y. et al., 2021	
Livestock farm water	Li et al., 2021f	−
Wastewaters	Li et al., 2020; Marathe et al., 2021	−
Sewages	Chen et al., 2019b; Mohsin et al., 2021	Peng et al., 2021; Wan et al., 2021
Urban sludge	−	Hirabayashi et al., 2021b
Wildlife (wild migratory birds)	Chen et al., 2019a, 2020; Cao et al., 2020; Fang et al., 2020; Ji et al., 2020	−
Water	Ji et al., 2020	−

*–, no available report.*

The drivers of these *tet*(X) genes in the isolates include promiscuous plasmids with various incompatibilities such as IncX1, IncQ1, IncW, IncA/C2, ColE2-like, IncFII, IncFIA, IncFIB, IncFIC, IncHIA, IncHIB, IncQ1-IncY hybrid, and many others, insertion sequences (IS*Vsa3*, IS*CR2*, IS*Aba1*, IS*26*, IS*4351*, and IS*1380*), transposon (Tn*As3*) and class one integrons (Bai et al., 2019; Sun C. et al., 2019; Sun J. et al., 2019; Li et al., 2020; Pan et al., 2020; Zheng et al., 2020; Soliman et al., 2021; Tang et al., 2021; Usui et al., 2021; Table 1). Of concern is that some *tet*(X)-bearing plasmids has become megaplasmid having acquired > 10 AMR genes conferring resistance against diverse classes of antimicrobial agents (Cui et al., 2020; Soliman et al., 2021; Sun et al., 2021a,b; Table 1). These megaplasmids poses a grave danger to the public and animal health as these plasmids are transmissible to other organisms and thereby jeopardizes antimicrobial therapy. Notably, a recent metagenomic study of human microbiome from 5 continents except Africa and Antarctica, showed that *Bacteroidaceae* has been an important reservoir and mutational incubator for mobile *tet*(X) orthologs in the human microbiome (Zhang et al., 2021). Furthermore, data mining from the NCBI database showed that mobile *tet*(X3/4/7) genes are already circulating in United States, South America (Colombia), Middle East (Qatar), Asia (Thailand and India), and Africa (Cote d’Ivoire) (Fang et al., 2020; Soliman et al., 2021; Wang et al., 2021f).

The recently discovered transmissible RND efflux pump gene, *tmexCD1-toprJ* also confers HLT resistance as well as resistance to antimicrobials in many other classes (Lv et al., 2020). Thus, it poses a threat to public and animal health. The ecological niches in which plasmid-mediated HLT resistance efflux pump gene have been detected are shown in Table 3. The *tmexCD1-toprJ1* was first variant of the mobile RND efflux pump gene discovered in China in 2019. It was carried by IncFIB, IncFII, and IncFIA plasmids in *Klebsiella* isolates from humans, food animals (chickens and pigs), meat and environment (sewage from food market and slaughterhouse environment) (Tables 2, 3). This occurrence in different ecological niches indicates that *tmexCD1-toprJ1* has disseminated to human, animal and environmental ecosystems and thus has One health ramifications. Other genetic elements driving *tmexCD1-toprJ* gene, including plasmids (IncX4, IncHI1B, IncQ, IncR, IncHI, plasmid hybrids, and others), insertion sequences (IS*26*, IS*903B*, IS*Kpn47*, IS*Kpn8*, and others), transposon Tn*5393*, and integrons have also been reported (He et al., 2021; Peng et al., 2021; Wan et al., 2021; Xu et al., 2022; Table 3). Very recently, variants of the plasmid-encoded transmissible RND efflux pump gene such as *tmexCD1-toprJ2* in *Rauoltella* isolate from a sick Chinese patient (Wang et al., 2021b) and *tmexCD1-toprJ3* in *Klebsiella* from urban sludge in Vietnam (Hirabayashi et al., 2021a) were reported. This distribution in different countries indicates that plasmid-mediated RND efflux mechanism of HLT resistance, is rapidly emerging and spreading in diverse ecological niches across the globe. A novel transmission pattern by which *tmexCD3-toprJ3* on integrative conjugative element (ICE) get integrated into other chromosomes of different bacterial hosts through cointegration of the circular intermediate was recently described (Wang et al., 2021e). Another transferrable novel efflux pump gene, *tnfxB3-tmexCD3-toprJ1b* cocarried with *tet*(X6) by ICE on the chromosome of *Proteus cibarius* and *Pseudmonas aeruginosa* isolates from chickens was also recently reported (Wang et al., 2021a). These findings point to the fact that ICE plays a huge role in the non-clonal restricted-dissemination of TIG resistance. So far, only Asian (Chinese and Vietnamese) studies have reported plasmid-encoded *tmexCD1-toprJ* (Table 3). It is worth noting that novel chromosomal-borne *tet*(L)_FSBL_ flanked by IS*257* and plasmid-encoded *tet*(L)_A117V_ efflux pump variants conferring resistance to TIG and eravacycline, were recently detected in *Staphylococcus* species isolated from pigs in China (Wang et al., 2021d).

**TABLE 3:** Ecological niches in which plasmid-mediated high-level tigecycline resistance efflux pump gene have been detected.

Country	Source of mobile tigecycline efflux pump gene-harboring organism	Mobile tigecycline efflux pump gene detected (organism)	Organism (sequence type)	Associated plasmid	Additional resistance factors	Contain genes encoding resistance against other last-resort antimicrobials	References
Vietnam	Humans and environment (urban sludge)	*tmexCD3- toprJ3* (*Klebsiella aerogenes*) and *tmexCD1-toprJ1* (*K*. *pneumoniae*)	ST4 and ST273	IncFIB, IncHI1B and IncC-IncX1 and IS*26*	*bla*_NDM–4_, *bla*_CTX–M–14_, *qnrS1*, *aac(6′)-lb-cr* and *cfr*	Yes	Hirabayashi et al., 2021a,b
China	Humans, food animal (chickens), food animal product (pork), and environment (chicken manure, chicken farm environment, slaughterhouse and sewage of food market)	*tmexCD1-toprJ1* (*K*. *pneumoniae* and *K*. *quasipneumoniae*) and *tmexCD2-toprJ2* (*Raoultella ornithinolytica* and *Klebsiella pnenumoniae*)	ST3447, ST37, ST1, ST11, ST180, ST236, ST1326, ST3332 ST967, ST147, ST896 and ST726 (*mrkABCDFHIJ*, *kfuABC*)	IncFIB, IncFII, IncX4, IncFIA, IncHI5 IncHI1B, and IncHI1B-FIB	***mcr*-1.1, *mcr*-8.1, *mcr*-8.5**, ***mcr*-10.1**, *bla*_DHA–1_, *bla*_CTX–M–3_, *bla*_IMP–4_, *bla*_NDM–1_, *bla*_OXY–1–3_, *bla*_CTX–M–15_, *bla*_TEM–1B_, *bla*_CTX–M–55_, *bla*_CTX–M–27_, *bla*_CTX–M–9_, *bla*_SHV–182_, *bla*_SHV–27_, *bla*_SHV–11_, *bla*_SHV–12_, *bla*_SHV–182_, *bla*_SHV–110_, *bla*_SHV–160_, *bla*_SHV–60_, *bla*_CTX–M–14_, *bla*_IMP–8_ *bla*_OXA–1_, *bla*_NDM–1_, *strA*, *strB*, *armA*, *aac(6′)-Ib-cr*, *aadA5*, *aac(6″) aac(6′)-Ib4*,*-IId*, *aph(3′)-Ia*, *aadA16*, *aac(3′)-IId*, *aph(6)-Id-cr*, *aph* *(6)-Id*, *aph(39)-IId*, *aph(39)-Ia*, *aph(3)-Ib*, *aac(3)-Iva*, *aph(6)-Ib*, *aph(4)-Ia*, *amrA*, *aph(3)-Ia*, *ant(3″)-Ia*, *aadA1*, *aadA2*, *aadA2b*, *arr3*, *ant(2″)-Ia*, *aph(39)-Ib*, *armA*, *aph(6)-* *Id*, *aph(39)-Ia*, *afrA27*, *mph*(E), *mph(A)*, *msr*(E), *cmlA1*, *catB*, *qnrB4*, *qnrS1*, *oqxA*, *oqxB*, *qnrS1*, *qnrB52*, *qnrB2*, *sul1*, *sul2*, *sul3*, *dfrA1*, *dfrA14*, *dfrA12*, *dfrA27*, *dfrB4*, *cfr*, *rmtB*, *floR*, *fosA*, *fosA6*, *tet*(A), *cmlA1*, *catB3*, *qacE*, IS*Ec28*, IS*Ec29*, IS*Ec59*, IS*903*, IS*CR1*, IS*1*, IS*26*, IS*903B*, IS*Kpn47*, IS*Kpn8*, Tn*5393*, Tn*As1*, *Int*1, *Int*2 and *rmtC*, metal resistance gene clusters (*silP-copG-cusA-cusB-cusF-cusC-cusR-cusS* and *copABCD*)	Yes	Lv et al., 2020; Sun S. et al., 2020; He et al., 2021; Peng et al., 2021; Qin et al., 2021; Wan et al., 2021; Wang et al., 2021b,g, 2022; Yang et al., 2021; Liu et al., 2022; Xiao et al., 2022; Xu et al., 2022

*ST, Warwick multilocus sequence type of Klebsiella isolates.*

Nevertheless, the NCBI database revealed that *tmexCD1-toprJ* gene is already spreading in Europe (United Kingdom), North America (United States), South America (Mexico), Africa (Kenya), and other parts of Asia (Pakistan and India) (Wang et al., 2021e). For now, the oldest isolate harboring plasmid-encoded *tmexCD1-toprJ* is a *Klebsiella pneumoniae* isolated in 2012 from the urine sample of a 21 year-old woman hospitalized for 21 days because of urinary tract infection (He et al., 2021). Therefore, mobile *tmexCD1-toprJ* likely emerged since at least a decade ago. But it seems to had remained restricted to *Klebsiella and Rauoltella* probably because it imposes a fitness cost, carried by plasmids with narrow host range or unstable in other *Enterobcaterales* (Lv et al., 2020). Nevertheless, the insertion sequence IS*26*, which likely transposed *tmexCD1-toprJ1* from chromosome (of *Pseudomonas*) to plasmid has the capacity to rapidly disperse *tmexCD1-toprJ* in *Enterobacterales* (Lv et al., 2020). The possession of both mobile *tet*(X) and RND efflux pumps could make diseases associated with such organism untreatable. Unfortunately, cocarriage of mobile *tet*(X) and *tmexCD3-toprJ3* has been detected (Hirabayashi et al., 2021b). High concentrations of *tmexCD1-toprJ1*, and *tet*(X3/4) was simultaneously detected in fecal samples of food-producing animals (Fu et al., 2021). Clonal dissemination and persistence of both *tet*(X) and RND efflux pumps gene is ensured since some of the MTR genes [e.g., *tet*(X3/4/5/6, *tmexCD1-toprJ1* and *tmexCD3-toprJ3*)] have been detected on chromosomes of bacteria (Chen et al., 2019a, 2020; Li et al., 2020; Liu et al., 2020; Peng et al., 2020; Zhang et al., 2020b; Wang et al., 2021e; Zheng et al., 2022). Both plasmid-borne and chromosomally-encoded *tet*(X) have been detected concurrently (Chen et al., 2020; Cheng et al., 2021a; Li et al., 2021a). Similarly, *tmexCD1-toprJ1* have also been simultaneously found on both plasmid and chromosome (Wang et al., 2021b).

More worrisome is that MTR tends to occur in conjunction with resistance to many other antimicrobial agents (including heavy metals), making the organism multi- to pandrug-resistant (Table 1). This coresistance further leads to limited options for therapy. Moreover, this situation can be exacerbated when an incriminated organism possesses an MTR mechanism [plasmid-encoded *tet*(X) or efflux pump] and gene coding resistance to other last-resort antimicrobials such as COL (conferred by *mcr*), fluoroquinolones, extended-spectrum cephalosporins and fosfomycin. Regrettably, such superbugs have already been isolated from humans (He et al., 2019; Ding et al., 2020; Ruan et al., 2020; Li et al., 2021a), food animals (He et al., 2019; Sun C. et al., 2019; He T. et al., 2020; Li et al., 2020, 2021c; Xu et al., 2021, 2022; Yu Y. et al., 2021), meat (Bai et al., 2019; Du et al., 2020; Mohsin et al., 2021; Soliman et al., 2021), and environment (Chen et al., 2019a; Cui et al., 2020; Mohsin et al., 2021; Yu Y. et al., 2021). Sadly, *tet*(X) even with genes coding resistance to other last-resort antibiotics, are horizontally/laterally transferred/acquired at a high frequency rate intra/inter-species/genus (Chen et al., 2019a; Sun C. et al., 2019; Cui et al., 2020; Ding et al., 2020; Du et al., 2020; Marathe et al., 2021; Soliman et al., 2021; Zhang et al., 2021). Likewise, the plasmid-encoded *tmexCD1-toprJ* with other resistance genes, including those encoding resistance to last-resort antimicrobials such as *mcr*, extended-spectrum β-lactamase and carbapenemase genes, has also been proven to be horizontally/laterally transferred at high frequency rate intra/inter-species/genera (Lv et al., 2020; Sun S. et al., 2020; Hirabayashi et al., 2021a; Qin et al., 2021; Wan et al., 2021; Table 3). Noteworthy, coexistence of *mcr*-1 and *tet*(X) genes, *tet*(X6), and *tet*(X7), on the same plasmid have been reported (Wang et al., 2021f; Xu et al., 2021). Also noteworthy, coexistence of HLT efflux pump genes (*tmexCD1-toprJ1* and *tmexCD2-toprJ2*) with carbapenem determinants (*bla*_IMP–4_, and *bla*_NDM–1_) on the same plasmid in clinical *Klebsiella* isolates have been reported (Wang et al., 2021h; Xiao et al., 2022). While conjugation (bacterial mating) is the major horizontal gene transfer (HGT) mechanism involved in the spread of MTR [*tet*(X) and HLT efflux pump] genes (Aminov, 2021; Cui et al., 2021a), transfer of these genes by other HGT mechanisms such as transformation (bacterial picking of naked DNA from environment) and transduction (prophage-mediated transfer) whereby viruses utilize the host’s (bacterial) genetic machinery for division and subsequently spreading the integrated genes to other infected bacteria have also been described (Cui et al., 2021a; Li et al., 2021f).

Conjugative plasmid-borne *tet*(X) is rapidly transferred and acquired to/from bacteria intra/interspecies/genera by conjugation as these plasmids are highly promiscuous mobile genetic elements (MGEs) possessing conjugation machineries like transposons, integrons and type IV secretion system (T4SS) that capture genes from the donor and readily releases it to recipient organisms (Virolle et al., 2020). However, some chromosomal mobile elements like ICEs and transposons, also contribute to the transfer of *tet*(X). For example, the mobility of chromosome-borne *tet*(X15) was associated with IS*Aba1*-bound composite transposon Tn*6866* (Li et al., 2021b,e,f), *tet*(X4) was flanked by IS*91* family transposase genes in chromosome of *Shewanella xiamensis* (Dao et al., 2022), and the location of transferable *tet*(X6) in ICE have also been reported (He D. et al., 2020; Wang et al., 2021c; Li et al., 2022). ICE are modular MGEs integrated into a host genome and are passively propagated during chromosomal replication and cell division. TIG use-selection pressure can induce the expression of *tet*(X) in ICE leading to excision and production of the conserved conjugation machinery (a T4SS), thereby possibly promoting genetic transfer to recipients (Zhang et al., 2020a). Moreover, MTR gene-bearing [especially *tet*(X)-bearing] plasmids are stably maintained and increase the growth rate of recipient organism (Cheng et al., 2021b), thus posing a challenge to antimicrobial therapy.

Remarkably, *tet*(X4)-plasmid remained stable in recipient organism for more than 100 generations in the presence of TIG selection pressure and up to 30 generations in the absence of TIG pressure (Li et al., 2021d). Thus, suggesting that elimination of MTR genes from the environment would be difficult if not practically impossible even after the withdrawal of TETs/TIG. The involvement of plasmid-encoded histone-like nucleoid-structuring (H-NS) protein in modulating the fitness of *tet*(X4)-bearing plasmids (such as IncX1) and ensuring the persistence of the gene have been reported (Cai et al., 2021). More critical is the situation where the transfer of *tet*(X4) resulted to increased size of transconjugant’s plasmid, thus, making it increasingly bigger than plasmids of the parental strain (Li et al., 2020, 2021d). This situation would suggest that the plasmid homologous recombination may occur in the recipient organisms following the acquisition of mobile *tet*(X). The homologous plasmid recombination (of IS*26*) has been speculated to involve the horizontal transfer (conjugation) of *tet*(X4) borne on a small non-self-transmissible plasmid possessing T4SS, whereby the small plasmid incorporated into bigger and broader-host plasmids that enabled *tet*(X4) transmission to recipient organism (Zhai et al., 2022). Notably, the *tet*(X4)-IncF family plasmids appear commonly observed in the form of hybrid plasmid (Du et al., 2020; Li et al., 2020; Sun C. et al., 2020; Zhang et al., 2020a; Zhai et al., 2022). This understanding may explain why *tet*(X4)—a gene proven to possess a high HGT frequency (Li et al., 2021c), appears to breach the biological boundaries and able to spread onto different bacterial genus and species. Feasibly, mobile *tet*(X) genes would be transferrable to other organisms and through diverse routes, thus posing a huge emerging threat to both clinical and empirical antimicrobial therapy.

Furthermore, the population structure of MTR gene-harboring organisms is diversified without clonal restriction. Lamentably, a considerable proportion of these organisms also harbor virulence-associated genes (VAGs), while some belong to the established virulent epidemic clones. For example, the mobile *tet*(X) was harbored by *E*. *coli* isolates belonging to zoonotic high-risk pandemic extraintestinal pathogenic *E*. *coli* (ExPEC) clones ST10, ST410, ST95, ST58, and ST167 (Manges et al., 2019; Sun C. et al., 2020; Li et al., 2021d; Marathe et al., 2021; Soliman et al., 2021; Tables 1, 3). Other pathogenic strains of *Enterobacterales* carrying mobile *tet*(X) such as *Proteus*, *Rauoltella* and *Acinetobacter* (Table 1) are major causes of infections especially in immunocompromised individuals (de Champs et al., 2000; Nakasone et al., 2015; Ayoub Moubareck and Hammoudi Halat, 2020). Essentially, virulent *K*. *pneumoniae* harboring mobile *tmexCD1-toprJ* isolated from microbial enumerations of hospitalized patients have been reported (Sun S. et al., 2020; He et al., 2021; Qin et al., 2021; Yang et al., 2021; Liu et al., 2022), particularly recovered from diverse ecological niches from different world regions (Tables 1, 3). Additionally, the presence of VAGs in the MTR gene-bearing strains could confer a fitness advantage to the organisms (Jiang et al., 2021).

## Causes of Mobile Tigecycline Resistance Development

Antibiotics are well established to induce selective pressure in the bacteria, which prompts them to acquire the resistance genes (Anyanwu et al., 2021a). Globally, besides being massively used as prophylactics, TETs serve as a growth enhancer and for metaphylaxis in livestock, including aquaculture sectors (Aminov, 2021). The older TETs have been overused in both human and veterinary settings worldwide for the past 60 years (Sun C. et al., 2019), especially in developing countries (Umar et al., 2021). Specifically, factors like the low cost, broad-spectrum activity against Gram-positive, Gram-negative, and atypical bacteria appears to contribute to this overuse of older TETs, together with their ability to be administered orally and intravenously (Li et al., 2020). Notably, the use of TETs as feed additive in Europe and United States was, respectively, banned in 2006 and 2017 (Aminov, 2013). The opposite appears to be so in the developing nations, where TETs remain among the over-the-counter (OTC) medications, procured without prescription by individuals, as well as administered to livestock. For instance, in China, TETs serve as antimicrobial agents mostly for prophylaxis, and growth enhancement in animal husbandry, from 2010 to 2015 with 2770 tons of TETs consumed in 2017 in livestock (He et al., 2019; Umar et al., 2021).

TETs are still being administered in sub-therapeutic doses for prophylaxis and growth enhancement, especially developing countries. Undoubtedly, such situation would be exerting selective pressure for developing TET-/TIG-r organisms. Counterfeit/substandard TETs appear to be scattered in the markets in developing countries, which contributes to exacerbate the challenges associated with the subtherapeutic antimicrobial concentration (Ayukekbong et al., 2017). For the reason that TIG has never been licensed/used in veterinary medicine/animal husbandry, the overuse of older-generation TETs would continue to strengthen the selective pressure, which would bring about the emergence of TIG resistance (Sun C. et al., 2019; Lv et al., 2020; Aminov, 2021; Umar et al., 2021). Some workers showed that clinical samples from a person who had consumed doxycycline but not received the TIG could bring about a mobile *tet*(X)-positive *E*. *coli* (Wang et al., 2020a). Therefore, increasing the clinical use of TIG in humans would contribute to the TIG selective pressure (Wang et al., 2019; Zhang et al., 2020a). Moreover, the consumption of other antimicrobials, particularly the phenicols, cephalosporins and fluoroquinolones, would equally exert such selective pressure for TIG resistance (Chen et al., 2020; Lv et al., 2020). Besides, the frequent administration of these antibiotics, which are usually administered in their subtherapeutic concentrations for prophylaxis and growth-enhancement in livestock, would potentially exert selective pressure. This would, therefore, prompt the rapid acquisition of resistance genes that possibly often involves the capture of MTR genes (especially by naturally competent organisms scavenging DNA fragments from the environment), by a single event of HGT. In fact, the use of florfenicol and tiamulin in livestock are believed to positively correlate with increasing concentration of mobile *tet*(X) and HLT efflux pump genes in animal fecal samples (Fu et al., 2021). Nevertheless, some resistance plasmids can capture resistance genes even in the absence of antibiotics selective pressure (Lopatkin et al., 2016).

## Possible Mobile Tigecycline Resistance Gene Transfer Mode and One Health Implication

AMR emerging from one ecosystem could easily move to another, thus having One Health ramifications (Anyanwu et al., 2020, 2021c). At the human-animal interface, individuals in close contact with animals such as animal handlers, animal health workers and owners/caretakers of animals, can easily acquire MTR. For example, *tet*(X3/4)-positive *E*. *coli* was isolated from the gut of live poultry market (LPM) workers and the LPM environment (Wang et al., 2019), suggesting that the workers possibly contracted the organisms following the handling of the birds, contaminated fomites or from environment. In fact, phylogenetic analysis revealed that *tet*(X4)-positive *E*. *coli* from human patients and animal origin were closely related indicating cross-sectorial clonal transmission of the organism between humans, animals and farm environment (Cui et al., 2021b). In addition, *tet*(X3/4) was present in the gut microbiomes of the LPM workers and surrounded environment (Wang et al., 2020a). The gut of humans and animals is conducive (“melting pot”) for the exchange of resistance genes by HGT (Shterzer and Mizrahi, 2015). Notably, the LPM workers worked with gloves and mask and did not take antibiotics for the previous 3 months (Wang et al., 2019), thus implying that improper removal of personal protective equipment (PPE) and inadequate infection prevention and control (IPC) practices like hand hygiene even after the use of PPE, could result in infection of humans by TIG-r organism from the animal environment. Thus, human-to-human transmission of MTR could occur following direct contact with individuals with poor basic IPC practices like hand hygiene or indirectly from them through contact with contaminated fomites such as farm equipment and farm workers’ paraphernalia. In essence, those in contact with animals (including veterinarians) are potential disseminators of MTR gene-containing organism into the public and veterinary hospital environments where these organisms have become a major nosocomial threat. Unfortunately, the organisms in ESKAPE (*Enterobacter*, *Staphylococcus*, *Klebsiella*, *Acinetobacter*, *Pseudomonas aeruginosa*, and *Escherichia coli*) group which are the most troubling pathogens associated with nosocomial and community infections worldwide (De Oliveira et al., 2020), have been reported as traffickers of MTR genes (He et al., 2019; Wang et al., 2019; Cui et al., 2020; Hsieh et al., 2021). However, the transmission of MTR at the human-animal interface is of more concern in developing countries because humans, especially those in rural areas, live in close contact with livestock (Alonso et al., 2017). Since *tet*(X3/4/5)-harboring organisms have been isolated from the gut of humans (Table 2), animals can be infected by TIG-r organisms following consumption of feed and/or drinking water contaminated (by feces) at the source by humans or by unhygienic animal caretakers and feed manufacturers. This mode of human to animal transmission of MTR is also more worrisome in developing countries because, they have poor environmental sanitation and many individuals/households have poor personal hygiene (Anyanwu et al., 2021b,c).

Since food animals have been shown to be reservoirs of MTR gene-harboring organisms (Tables 1–3), they are potential source for farm-to-plate transmission of MTR even in human population without antimicrobial use. For example, *tet*(X4)-positive Enterobacteriaceae was isolated from feces of healthy individuals who had never taken antibiotics (Ding et al., 2020; Ruan et al., 2020), indicating a possible acquisition from livestock/food animal products and circulation within the community. Slaughterhouses are critical points of contamination of animal products with MTR gene-harboring bacteria. For example, *tet*(X4)-positive *E*. *coli* and *tmexCD1-toprJ1*-positive *Klebsiella pneumoniae*/*Proteus mirabilis* have been isolated from slaughterhouses/slaughterhouse wastewaters (Sun C. et al., 2019; Li et al., 2020; Mohsin et al., 2021) and meats (pork, beef, chicken, and duck meat) for human consumption (Bai et al., 2019; Du et al., 2020; Mohsin et al., 2021). Thus, handling and consumption of raw/half-cooked meat/associated products is a route for the acquisition of TIG-r bacteria. It also means that individuals working in slaughterhouses can acquire these organisms from the slaughterhouse environment and contaminated fomites. Meat contamination by resistant organism is easy in developing countries due to unhygienic slaughtering methods and an unsanitary slaughterhouse environment (Anyanwu et al., 2021a). For instance, high prevalence of TIG and COL resistance has been reported in humans in a region with no history of COL/TIG use in Nigeria (Ugah and Udeani, 2021). Thus, indicating the acquisition from livestock, possibly through contact and/or farm-to-plate transmission since TETs and COL are used massively in livestock in Nigeria (Anyanwu et al., 2021b).

Since healthy and sick human fecal and clinical samples yielded *tet*(X3/4)-positive *E*. *coli* and *Acinetobacter* and *tmexCD1-toprJ1*-positive *Klebsiella* (He et al., 2019; Wang et al., 2019; Ding et al., 2020; Zeng et al., 2021), and these organisms were also recovered from human sewage (Cui et al., 2020), wastewaters (Marathe et al., 2021), animal feces (Sun C. et al., 2019; Cui et al., 2020; Li et al., 2020; Yu Y. et al., 2021) and slaughterhouse wastewaters/sewage (Mohsin et al., 2021), TIG-r organism/MTR can enter environmental ecosystem through anthropogenic (domestic, laboratory, and hospital) and agricultural (farm and slaughterhouse) sewages and wastewaters (Table 2). Asides improper disposal of wastes into environmental surface waters, rainfall run-offs could carry TIG-r organisms from wastes/sewages/sewage-spills on land/landfills into these surface waters. For example, TIG-r *E*. *coli* carrying *mcr*-1 gene was recovered from sea water polluted with anthropogenic (domestic, hospital, and industrial wastes) and agricultural wastes in Algeria (Drali et al., 2018). Animals (such as straying, scavenging, wild/urban animals like water birds and pigeons, amphibians, snails, and so on) that depend on environmental surface waters for sustenance, could easily acquire these organisms. For example, *tet*(X3)-positive *Acinetobacter* has been isolated from waterfowls (geese and ducks) and pigeons and their neighboring environment (Cui et al., 2020). Waterfowls especially ducks, have been shown to be the natural host of the bacterium *Riemeriella anatipestifer*—the ancestral source/reservoir of diverse *tet*(X) genes (Umar et al., 2021; Zhang et al., 2021; Li et al., 2022). China has the largest waterfowl breeding industry in the world, and massive antibiotics (especially TET) is inevitably being used in the breeding process (Cui et al., 2021b). Large-scale use of TETs in the Chinese waterfowl breeding industry (partly to prevent septicemia *anserum exsudativa*—an economically important diseases caused by *Riemerilla anatipestifer*) is possibly the major cause of emergence and spread of MTR in China (Aminov, 2021; Cui et al., 2021b; Umar et al., 2021). Environmental surface water pollution is also of more concern in developing countries because, in most of these nations, poor environmental sanitation with practices like improper disposal of human/agricultural sewage, human open air defeacation and nomadic animal rearing allowing animals to discharge feces indiscriminately on land and surface waters, are still common (Anyanwu et al., 2021a,c). Furthermore, straying and scavenging animals, especially dogs/cats, are common in most developing countries. These companion animals can acquire TIG-r organisms from improperly disposed-off human excreta through coprophagic behavior (Leite-Martins et al., 2015) and then transfer the organisms to their caretakers.

Slaughterhouse wastes such as animal manure and internal organs are often used as economical mode in feeding of fish and companion carnivores (dogs and cats) in developing nations (Anyanwu et al., 2021c). Thus, a potential route through which TIG-r bacteria can enter aquaculture and companion animals. Furthermore, integrated farms common in developing countries, could facilitate the exchange of TIG-r organisms between food animals and aquaculture since excretions from livestock that may contain TIG-r organisms/MTR gene-containing organisms and un-metabolized antimicrobials, serve as food to fish that receives little or no supplementation (Anyanwu et al., 2020). There is also a possibility that TIG-r organism in anthropogenic/agricultural sewage/wastewater could escape treatment and get discharged into surface waters or end up on land/farm soil as organic manure. Escape of TIG-r organisms from sewage/wastewater treatment plants (WWTPs) into surface waters could lead to infection/colonization of feral aquatic animals/aquaculture (Cherak et al., 2021). For example, *tet*(X4)-positive *E*. *coli* has been isolated from shrimps captured for human consumption (Li et al., 2019). Thus, coming in contact with and consumption of contaminated/colonized aquatic foods, and the use of aquatic animals like fish/shrimps in making fish meal (which serves as protein source in livestock feed) is a route by which MTR can enter human and animal ecosystems from the aquaculture sector. Infected/colonized persons could release these organisms into the environment (land/soil) even in surface waters during swimming and bathing as well as from ships. Unfortunately, in developing countries, these waters are used (especially by those living at coastal areas) for various purposes (such as cooking, bathing, laundry, processing of food, etc.) potentially creating opportunities for the exchange of TIG-r organisms between humans and aquatic environments (Anyanwu et al., 2021c). Moreover, the water currents would also serve as the transport of these dangerous organisms to different parts of the world (Anyanwu et al., 2020).

Human/agricultural sewage and animal manure used as organic fertilizer on land, are a potential source for the dissemination of TIG-r organisms/MTR into soil and botanical (plant) ecosystems (Sun J. et al., 2019). For example, *tet*(X3/4)-harboring organisms (*E*. *coli* and *Acinetobacter*) and high abundance of these genes as well as *tmexCD1-toprJ1*, have been detected in farm soil and soil within livestock farms (Sun J. et al., 2019; Cao et al., 2020; Li et al., 2020; Cui et al., 2021b; Yu Y. et al., 2021). Garbage/synathropic flies that feed/breed on animal manure can pick these organisms from the soil and transport them to other ecological niches. Rainfall drops could raise TIG-r organisms from the soil to contaminate/colonize plants from where the MTR is re-incorporated into the food chain. Individuals who handle and eat plant products (fruits, vegetables), especially when raw, are likely to be infected by these organisms (Anyanwu et al., 2020). Grazing animals could easily acquire resistant organisms from contaminated plants (grasses and herbages). Encouragingly, composting or anaerobic digestion of animal manure before use as organic fertilizer in agricultural farms reduces the dissemination of AMR from animal to environmental ecosystem (Gao et al., 2019). It is worth noting that the soil contains TET-inactivating enzymes that detoxify only naturally occurring first-generation TETs, such as chlortetracycline and oxytetracycline, but fail to oxidize D-ring substituted analogs, including TIG, eravacycline and omadacycline (Gasparrini et al., 2020).

**FIGURE 1:**
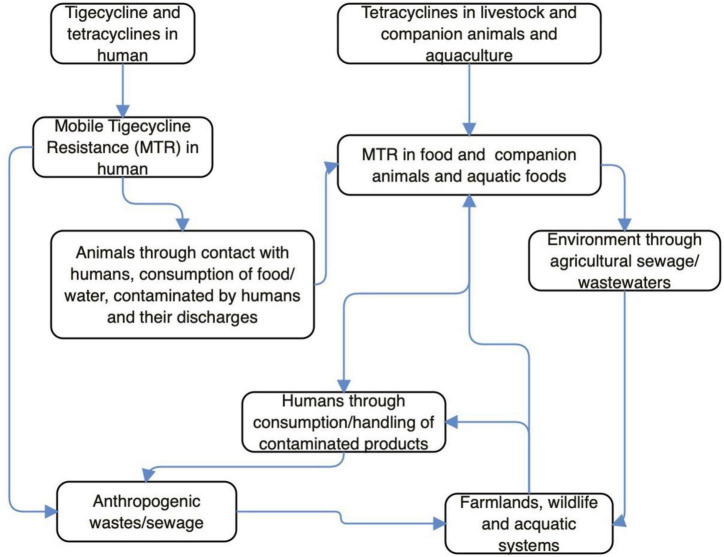
Potential pathways through which mobile tigecycline resistance (MTR) emerges and builds up in human, animal, and environmental ecosystems.

Antibiotics are not used in wildlife, but sadly, *tet*(X3/4/5)-positive *E*. *coli* and *Acinetobacter* has been isolated from wild birds (Chen et al., 2019a, 2020; Cao et al., 2020; Mohsin et al., 2021). This implies that MTR has already disseminated into the wild and it is of anthropogenic/agricultural origin. Wild animals can acquire TIG-r organisms/MTR following contact with anthropogenic (market, farm, slaughterhouse, home, and laboratory) wastes/sewages, drinking of contaminated water/wastewaters in the wild or environment with anthropogenic activity, feeding on carrion, and consumption of lower animals/plant products (fruits, vegetable, and grasses) contaminated by insects (on perching) or excreta of overflying birds (Anyanwu et al., 2020). The *tmexCD1-toprJ1* and mobile *tet*(X) genes have been detected in isolates from sewage of food market environment and slaughterhouses (Mohsin et al., 2021; Wan et al., 2021). Therefore, wild mammals/birds could easily acquire MTR from these ecological niches. Wildlife infected/colonized by TIG-r bacteria could disseminate these organisms to places (markets, playing grounds, etc.) frequented by humans (Anyanwu et al., 2020). Since *tet*(X3/4/5)-positive Enterobacteriaceae has been recovered from wild migratory birds (Cao et al., 2020; Tables 1, 2), it means that migratory wild birds can transport MTR (even following discharge of organisms in water bodies) from one country/continent to another (Anyanwu et al., 2020; Cao et al., 2020; Wan et al., 2021). Migratory wild birds always live far from dense human population and are not exposed to antibiotics directly; however, they can have access to animal and animal waste containing TIG-r organism/MTR genes (Cao et al., 2020; Umar et al., 2021). Thus, the presence of resistance gene in these birds is evidence of anthropization (Ewbank et al., 2021). The illustration of potential pathways through which MTR emerges and builds up in human, animal and environmental ecosystems can be seen in Figure 1. Besides tigecycline and tetracycline moving via human, livestock/companion animals and aquaculture, the MTR in both above mentioned two scenarios would combine and penetrate into the environment through agricultural sewage/wastewater. This eventually cycles back to human again, whether anthropogenically and or farmland/aquaculture systems. In other words, through the consumption/handling of contaminated products, the agricultural sewage, farmlands/wildlife/aquatic systems, anthropogenic wastes/sewage/wastewater and humans strengthen the MTR’s existence within the environment.

As the world is becoming one village, TIG-r organisms can be transported from one location to another through international/domestic food animal/meat/wildlife trade and travel (Bai et al., 2019; Wang et al., 2020b; Wan et al., 2021). Considering the fact that some of the MTR gene-containing isolates (especially *E*. *coli*, *Klebsiella*, and *Acinetobacter baumanii*) belonged to the high-risk zoonotic pandemic extraintestinal pathogenic multiresistant clones and contains virulence genes (Bai et al., 2019; Sun J. et al., 2019; Marathe et al., 2021; Qin et al., 2021; Tables 1, 3), there could be outbreak of difficult-to-treat or untreatable diseases with outrageous health and economic impacts to the human and animal sectors. Already, virulent multidrug-resistant *tet*(X5)-harboring organisms have been isolated from sick animals (Chen et al., 2020). Notably, MTR is a monumental threat to global food safety/security and public health. Lamentably, the treatment of diseases associated with MTR is difficult due to limited/unavailable therapeutic option (He et al., 2019).

## Mobile Tigecycline Resistance Spread and Mitigating Strategies

Globally, MTR has an immense One Health ramification and therefore warrants urgent intervention. Since older-generation TETs have been used for a long time, routine testing of isolates from diverse sectors (humans, animals—food and companion, environment, and aquaculture) for TIG resistance, is urgently warranted (Chen et al., 2019a, 2020; Yongchang et al., 2019). Retrospective screening (if possible, by whole genome sequencing) of archived TET-resistant/TIG-resistant isolates for plasmid-encoded *tet*(X) and *tmexCD1-toprJ* genes, is important to understand the evolution and spread of MTR. Metagenomic studies could be useful in determining the magnitude of MTR mechanisms in different ecological niches (Fang et al., 2020). There is a need for developing affordable rapid test kits capable of detecting the diverse MTR genes, including those genes yet to emerge (Fang et al., 2020). A consideration should be given for pre-slaughter examination of food animals for MTR and post-slaughter testing of processed meat/associated products prior to distribution to retail shops, consumers or export. Quarantine and examination of individuals returning from TET/TIG resistance/*tet*(X)/*tmexCD1-toprJ* endemic regions and imported food/companion/wild animals capable of serving as vectors for the transportation of *tet(*X)/*tmexCD1-toprJ* gene-containing organisms. The decolonization of carriers of these organisms/genes is important to reduce their spread from one place to another. Intensified global surveillance (involving and supporting TIG resistance researches in developing countries) of MTR in diverse ecosystems is urgently needed to devise a holistic One Health approach in curtailing the spread of MTR before it gets out of hand (Okpala et al., 2021).

As new effective antimicrobial agent that is capable of destroying superbugs is not yet available, there is need for increased use of non-antibiotic agents such as antimicrobial peptides and vaccines in order to reduce the use of antibiotics and development of MTR. Rational antimicrobial use should be promoted as this will inhibit the formation and evolution of cointegrate plasmids harboring emerging novel resistance genes (such as *mcr* and MTR genes) thereby reducing the transmission of these genes among bacteria (Lu et al., 2021). Hence, continuous surveillance of the emergence of *mcr*-carrying MDR plasmids and MTR gene-bearing MDR plasmids in bacterial isolates is warranted (Lu et al., 2021). The sale and use of TETs and florfenicol, especially in developing countries, should urgently be reconsidered (Peng et al., 2021; Wang et al., 2021b). For now, TIG is not available in most developing countries. Strict regulation is crucial to prevent the flooding of markets in these nations with counterfeit TIG. This salvage antibiotic should be strictly guarded by not licensing it for use in veterinary medicine. Finally, there is an urgent need for countries to improve on their antimicrobial stewardship emphasizing massive education of the stakeholders in human and animal medicine (physicians, veterinarians and allied health workers, policy makers, and clinical microbiologists) and the public, on the dangers associated with MTR as well as basic IPC practices like hand hygiene (which breaks transmission cycle of resistant organisms/pathogens), and environmental sanitation (Wan et al., 2021). It is also crucial to lay emphasis on prompt/adequate diagnosis and antimicrobial susceptibility testing as some of the isolates in this review showed susceptibility to some available antibiotics (Hsieh et al., 2021).

## Concluding Remarks

In this current work, we have reviewed why MTR is an emerging health catastrophe requiring urgent One Health global intervention. We have discussed the antimicrobial activity of TIG and its mechanism of resistance. We have also discussed the distribution, reservoirs, and traits of MTR gene-harboring isolates, and the causes of MTR development. It is important to reiterate that the possible MTR gene transfer mode and its One Health implication, is very important, prior to understanding MTR spread, and how to mitigate it. The fact that MTR threatens the clinical efficacy of the salvage antibiotic, there is need to understand the position of TIG usage in treating deadly infections in humans, especially those that have been caused by superbugs, including carbapenem- and colistin-resistant bacteria. It is key to emphasize that TIG despite being a third-generation TET (belonging to glycycline) antibiotic, has never been used in animal husbandry anywhere around the globe. The regulation on the use of older-generation TETs particularly in developed countries, further demonstrates why this issue is very important. Some promising areas that provide the basis for further investigations in the future were identified in this review. More so, the danger posed by the rapidly emerging plasmid-mediated HLT resistance cannot be overemphasized. Thus, the global community needs to rise, to improve their antimicrobial stewardship and halt the injudicious use of antimicrobial agents especially TETs/TIG, to protect human and animal health.

**FIGURE 2:**
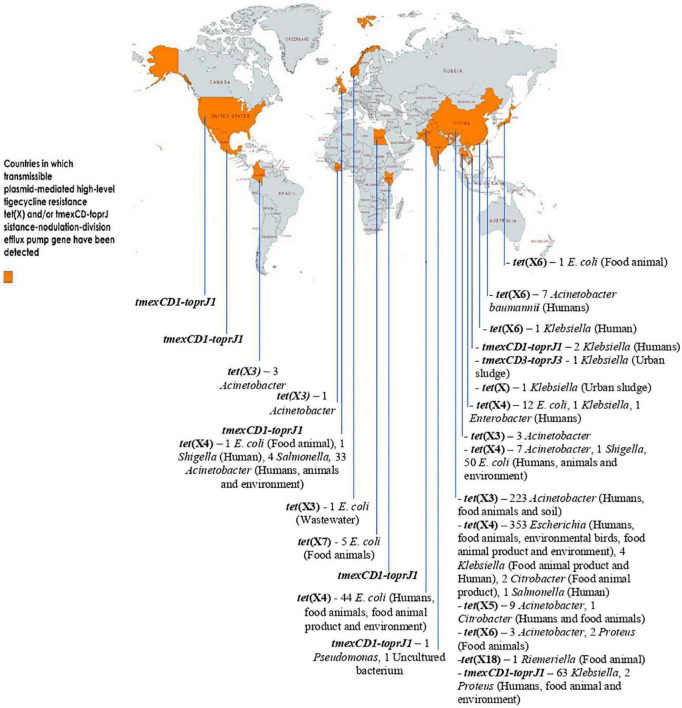
Distribution of plasmid-encoded high-level tigecycline resistance gene-harboring organisms in diverse ecological niches worldwide. The data set for some of the genes (with or without ecological niches) was based on data mining from the NCBI database by Fang et al. (2020), Peng et al. (2021), and Wang et al. (2021e) This map was created using an online service (https://mapchart.net/).

Due to the overuse of TETs/other antimicrobial agents in human and animal sector as well as inappropriate use of TIG in humans, especially in developing countries, a fast-spreading MTR mediated by horizontally/laterally transmissible *tet*(X)- and *tmexCD1-toprJ* RND efflux pump gene-bearing plasmids, are jeopardizing the clinical usefulness of the last-ditch antibiotic TIG. For now, *tet*(X3) to *tet*(X7), and *tet*(X18) are the plasmid-encoded HLT resistance *tet*(X) gene variants known. Others such as *tet*(X14) and *tet*(X15) that also confer HLT resistance are chromosome-borne yet mobilizable by ICEs and composite transposons. Plasmid-borne *tet*(X) genes are harbored by a diversity of bacteria in different families but predominantly *Enterobacterales*. Three variants of *tmexCD-toprJ* RND efflux pump gene (*tmexCD1-toprJ1*, *tmexCD2-toprJ2*, and *tmexCD3-toprJ3*) with specific host range and limited plasmid vector have been described. Importantly, the distribution of plasmid-encoded HLT resistance gene-harboring organisms in diverse ecological niches worldwide is depicted in Figure 2. Clearly, the MTR appears strongly emerging and spreading rapidly in the human, animal and environmental ecosystems, including wildlife, worldwide. Thus, MTR is a potential health catastrophe in the making, if aggressive efforts to curb its spread is not implemented immediately. An aggressive One Health surveillance/monitoring and intervention approach to tackle this rapidly emerging global health threat, is urgently warranted.

## Author Contributions

MUA and OCN: conceptualization, methodology, investigation, formal analysis, supervision, funding acquisition, and writing—original draft. CORO and IME: formal analysis, visualization, resources, writing—review and editing, and project administration. All authors contributed to the intellectual content and agreed to the final submitted version.

## Conflict of Interest

The authors declare that the research was conducted in the absence of any commercial or financial relationships that could be construed as a potential conflict of interest.

## Publisher’s Note

All claims expressed in this article are solely those of the authors and do not necessarily represent those of their affiliated organizations, or those of the publisher, the editors and the reviewers. Any product that may be evaluated in this article, or claim that may be made by its manufacturer, is not guaranteed or endorsed by the publisher.
